# Ablation of Refractory Papillary Muscle Ventricular Tachycardia Warranting Multiple Adjunctive Ablation Techniques: A Combined Approach for Success

**DOI:** 10.19102/icrm.2020.110206

**Published:** 2020-02-15

**Authors:** Mark Pollet, Ben Jenny, Adwait Mehta, Austin Howard, Nilesh Mathuria

**Affiliations:** ^1^Division of Cardiac Electrophysiology, Texas Heart Institute, Houston, TX, USA

**Keywords:** Ablation, papillary muscle, ventricular tachycardia

## Abstract

A 27-year-old male presented to our institution with recurrent unifocal premature ventricular contraction/nonsustained ventricular tachycardia (VT) with associated cardiomyopathy. The patient had undergone three prior ablation procedures with continued arrhythmia. Mapping led to identification of the VT arising from the basal aspect of the left ventricular anterolateral papillary muscle. Conventional ablation techniques were unsuccessful. We incorporated adjunctive ablation techniques in this case that ultimately led to a successful outcome. The present discussion covers the roles of intracardiac echocardiography, induced apnea, and low-ionic irrigation.

## Introduction

Despite improvements in catheter technology and electroanatomic mapping for ventricular tachycardia (VT) ablation, achieving desired ablation outcomes remains difficult in certain locations due to anatomical constraints among sites such as the left ventricular (LV) summit, intraseptal VT, and papillary muscles (PMs). Understanding the biophysics of radiofrequency (RF) ablation is critical for optimizing successful outcomes. The distribution of current and the focal point of temperature rise are dependent on multiple catheter-related factors including time length of contact, force of contact, power, and irrigation. We present a case of a resistant, idiopathic, left VT located at the base of the anterolateral PM (ALPM). Furthermore, we discussed the incorporation of adjunctive ablation strategies such as intracardiac ultrasound, ventilation suppression, and reduced ion [half-normal saline [(HNS)] irrigation to facilitate successful ablation.

## Case study

A 27-year-old male with a significant burden (30%–50%) of premature ventricular contractions (PVCs) presented for ablation at our institution. During the preceding 10 years, he had undergone three ablation attempts including one epicardial approach that suppressed the PVCs for two hours. The patient had also developed a cardiomyopathy (ejection fraction: 35%) with LV dilatation (6.0 cm), which was felt to be a result of the high burden of ventricular ectopy.

**Figure 1:**
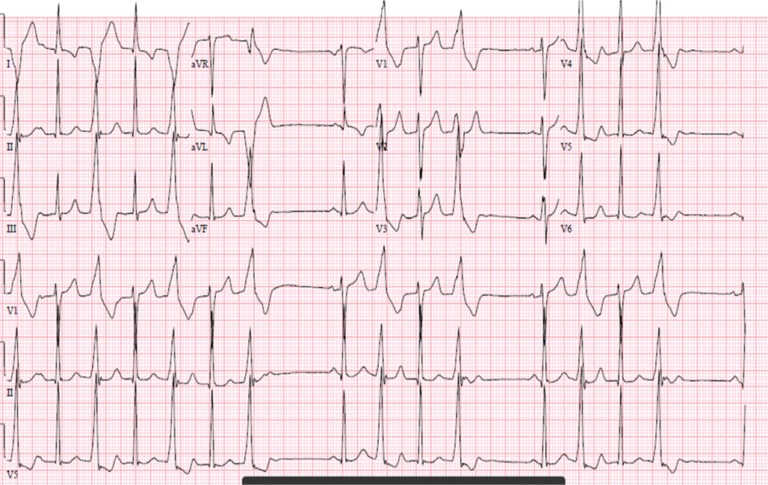
Electrocardiogram morphology of PVC.

At baseline, the patient had runs of bigeminal and trigeminal PVCs despite medical therapy with beta-blockers and amiodarone at the time of the current presentation **(Figure 1)**. Prior medical therapy included dronedarone; flecainide; and, currently, amiodarone, without any change in PVC burden. The PVC had a right bundloid morphology with an inferior axis and a sharply negative initial deflection in leads I and aVL, suggesting a lateral left ventricular origin. Because of the relatively delayed onset-to-peak deflection seen prominently in leads V1 and I, the need for an epicardial approach was considered. We, therefore, prepared to convert to epicardial access if our initial endocardial evaluation was insufficient.

The patient was fully sedated with general endotracheal intubation and vascular access was obtained via bilateral femoral venous sheaths and a single femoral artery sheath. An intracardiac echocardiography (ICE) catheter was placed in the right atrium and the relevant ventricular anatomy was surveyed. Next, a decapolar sensing catheter was placed in the coronary sinus and a quadripolar sensing catheter was placed in the right ventricular apex. Finally, a steerable, force-sensitive, irrigated ablation catheter was advanced retrogradely across the aortic valve into the LV, and heparin was given to achieve a therapeutic activated clotting time of 300 seconds to 350 seconds.

**Figure 2:**
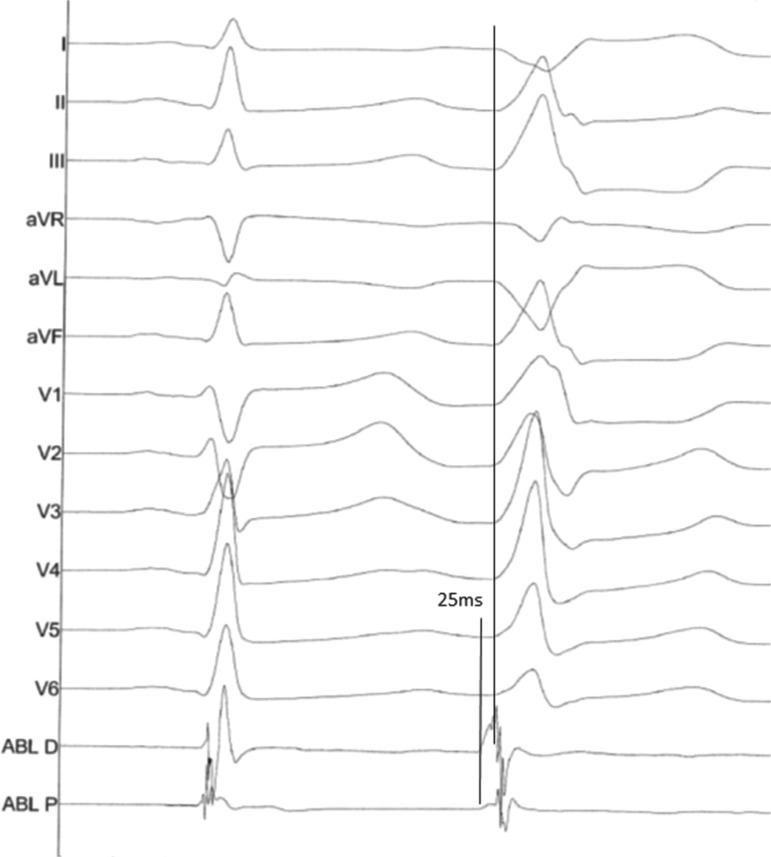
Earliest site during mapping of PVC.

**Figure 3:**
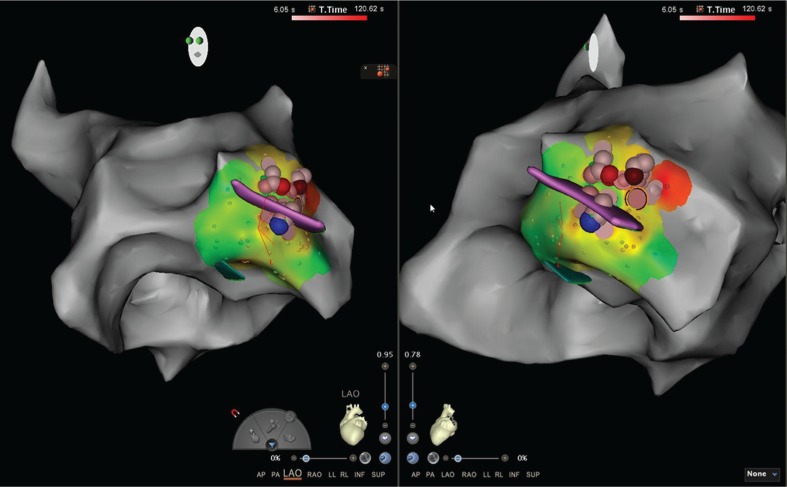
Electroanatomic mapping of clinical PVC: left anterior oblique and left posterior oblique views. Earliest site of activation in the small region of the ALPM. Red: site of successful ablation. Maroon/blue dots: manual tags with areas of fractionation likely resulting from prior ablation attempts. The majority of lesions in the region lasted less than 10 seconds in duration (peach-colored points) due to catheter instability.

**Figure 4:**
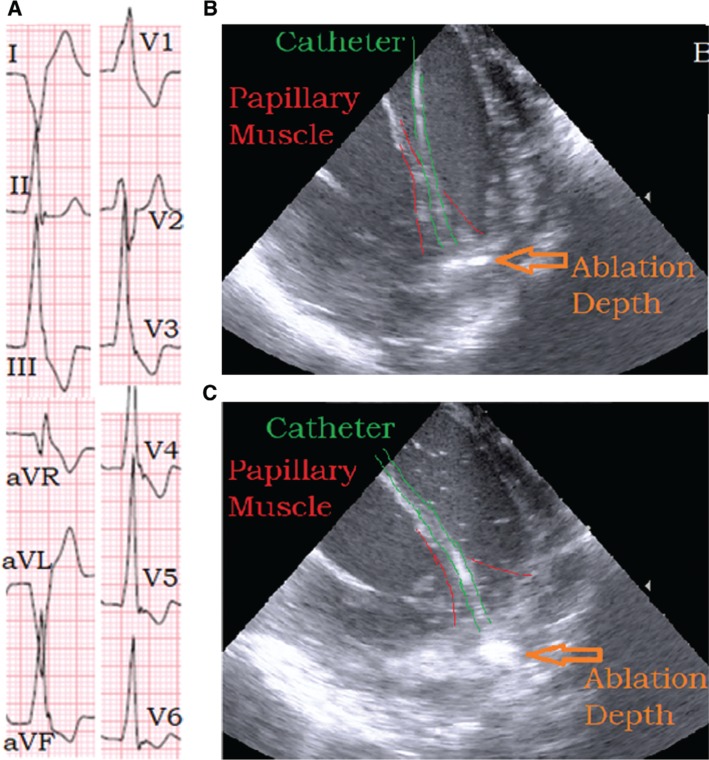
**A:** Morphology of the PVC. **B:** Ablation catheter placed at the base of the ALPM postablation with NS. **C:** Ablation catheter located at the base of the ALPM after ablation with HNS. Further penetration into the myocardium as seen by the highly echodense region deep to the endocardial contact point of the ablation catheter tip.

Using a combination of ICE imaging and an electroanatomical mapping system, the earliest signal for the PVCs was identified at the base of the ALPM with a local electrical signal seen at 25 ms pre-QRS **(Figure 2)**. Electroanatomic mapping revealed a small region of early electrograms 15 ms to 25 ms pre-QRS over the ALPM **(Figure 3)**. There was difficulty in maintaining catheter contact on the region of interest. Extensive ablation at this site with normal saline (NS) irrigation and a maximum power of 50 W yielded temporary suppression of the PVCs **(Figure 4B)**. In fact, the PVCs were suppressed for a full 60 minutes of observation but returned during the washout period of an isoproterenol challenge.

At this point, we held a discussion regarding planning for epicardial access versus switching to high-impedance irrigation such as HNS. We elected to attempt the latter HNS method because it offered an endocardial option. An epicardial approach was less desirable, as the patient had already been therapeutically anticoagulated and a prior epicardial attempt was unsuccessful.

**Figure 5:**
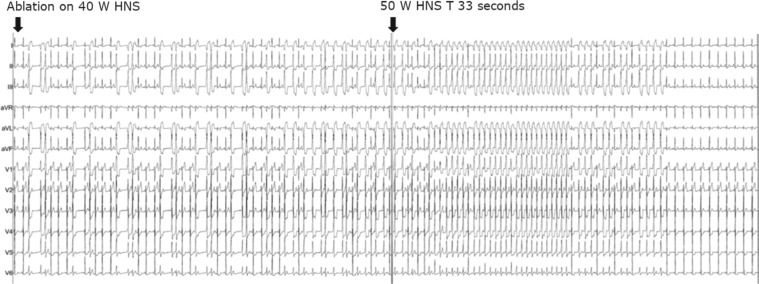
Sudden ectopy with resolution of VT after increasing the power to 50 W using HNS irrigation along with induced apnea.

A transseptal puncture was performed using an 8.5-French Mullins sheath (Abbott Laboratories, Chicago, IL, USA) and the ablation catheter was again moved to the base of the PM. With this configuration, we were able to obtain images by ICE, which proved contact and stability of the ablation catheter at the base of the PM **(Figure 4C)**. However, despite these maneuvers, the ablation catheter continued to have difficulty in maintaining stability over the desired location. Electroanatomic data also confirmed the time of each attempted ablation lesion frequently to be less than 10 seconds **(Figure 3)**. To minimize respirophasic force contact variations, we induced apnea through anesthesia support. We then performed the ablation with HNS initially at 40 W and subsequently at a maximum power of 50 W for 30 seconds into the lesion. Immediately after titrating to 50 W, a brief run of slow VT occurred, after which time, no further PVCs were seen **(Figure 5)**. We continued this ablation for a total of 120 seconds. There was no evidence of steam pops or any sudden rise in impedance. Based on the immediate clinical response at 50 W versus 40 W, it appeared that the induced apnea, catheter stability guided via ICE, and use of higher power (50 W) with HNS were all required for successful VT elimination.

After the final ablation, PVCs were no longer observed over the course of an observation period. The patient was observed for 48 hours prior to discharge. Amiodarone was subsequently discontinued. The patient participated in clinical follow-up visits at one, two, and six months postablation; at six months, he showed an improvement in LV ejection fraction (56%) and LV dilatation (5.0 cm) as well as complete resolution of the previously seen PVCs according to 24-hour ambulatory electrocardiogram monitoring.

## Discussion

The present case highlights the complex nature of PM VT given the variable anatomy, difficult catheter stability, and often intramural/midmyocardial site of origin of the ectopy. Ultimately, the present case required the use of multiple adjunctive ablation techniques beyond typical activation mapping, which included real-time ICE, controlled apnea, and low-ionic irrigation along with high power during catheter ablation.

RF catheter ablation has become a standard treatment for supraventricular tachycardia and VT. Historically, the use of RF ablation in the setting of VT was limited by the inability to deliver lesions deep enough within the myocardium to reach the arrhythmogenic focus.^1^ Lesion size is limited by the temperature at the electrode–myocardial tissue interface. As this temperature approaches 100°C, denatured proteins and boiling plasma form a coagulum around the catheter tip, which decreases the effective surface area and increases impedance, ultimately reducing the amount of current delivery into the tissue. One method to increase lesion size, first proposed by Wittkampf and Hauer in 1988, used irrigated ablation catheters.^2^ Irrigated-tip catheters mitigate the challenges related to heating of the blood pool by reducing the temperature at the electrode–tissue interface. This avoids the formation of coagulum and subsequent impedance rise, which facilitates target RF current delivery into the tissue.^1^ This has been shown to enable enhanced energy delivery into the target tissue and results in an increase in lesion size as compared with when using a nonirrigated catheter.^3,4^

There have been numerous investigations to better understand the characteristics of catheter irrigants and their effects on the parameters of ablation lesions to date. Similar results have been noted using chilled versus room-temperature NS.^4^ In 2004, Petersen et al. published their data from both in vitro and in vivo animal models using differing concentrations of saline and dextrose to vary impedance at the electrode–tissue interface. Their findings found no major difference in the lesions produced by distilled water, dextrose, and NS.^5^ In addition, however, they found that smaller lesions were produced when the catheter was irrigated with 3% and 5% saline solutions. This led to the conclusion that increased electrical conductivity of the irrigant reduced lesion size by dispersing RF energy away from the tissue, which was the path of higher impedance in these instances. The idea that electrical conductivity could alter lesion size was further demonstrated by Nguyen et al., who investigated the use of HNS as an irrigant—that is, a liquid with intermediate conductive properties between dextrose and NS.^6^ These authors found larger lesions were produced with HNS and dextrose versus NS. Further, there was no increase in steam pops in the HNS group, yet an increase in such was noted with dextrose. Given the potential safety profile and availability of HNS, further evaluation of HNS was performed in an ex vivo model and the results compared with bipolar RF energy. Consistent with prior publications, unipolar RF with HNS led to the creation of larger lesions when compared with unipolar RF with NS. Further, when comparing bipolar RF with NS to sequential unipolar RF with HNS, a similar lesion volume was noted. Bipolar RF, however, did create a statistically significant deeper lesion, albeit with a small absolute difference (13 ± 1.4 versus 12.5 ± 0.5 mm; p = 0.02). These findings suggest that HNS could be considered in cases where traditional unipolar RF is insufficient, able to crease similar lesions to those of bipolar RF.^7^ A subsequent human trial using HNS in 94 cases of VT refractory to standard RF ablation was recently described. In this prospective multicenter trial of patients who failed conventional ablation, 83% achieved acute success with HNS administration. All sites ablated were felt to be midmyocardial and/or presenting a complex substrate warranting a deeper lesion set. The majority of HNS lesions were located in the interventricular septum/LV summit, whereas other cases involved sites such as the PM (13%), LV free wall (15%), and RV (16%). Further, 12% developed steam pops during RF, with no acute complications. This led to the conclusion that HNS is safe and effective when delivering high-power RF lesions for deep myocardial penetration.^8^ Success has also been reported for PVCs arising from the LV summit in cases where HNS was used after multiple previous failed attempts.^9^ Dextrose has also been successfully applied via the great cardiac vein to ablate VT in a previously unsuccessful case, and the suggestion has been made that the use of low- or nonionic irrigants may be useful as an adjunct after failed ablation involving conventional NS.^10^

Because RF catheter contact has a significant impact on lesion formation, cardiac and respiratory motions have important implications on procedural success.^11^ The integration of detailed three-dimensional (3D) cardiac models obtained by computerized tomography or magnetic resonance imaging with 3D electroanatomic mapping and, recently, with fluoroscopic imaging enables precise anatomical catheter navigation under both nonfluoroscopic and fluoroscopic guidance. These strategies seek to improve the operator’s understanding of the catheter’s location by showing it in relation to a detailed 3D model of the cardiac anatomy.^12,13^

The cardiac anatomy can be viewed in real time using ICE. ICE has emerged as a complementary technique to standard fluoroscopic imaging and an ideal modality for imaging structures highly relevant to catheter ablation procedures.^14^ In addition to providing intraprocedural guidance during transseptal catheterization,^15^ phased-array ICE imaging has been very useful for understanding catheter placement, contact, and movement as well as in determining cardiac wall thickness and proximity to neighboring structures. In some cases, ICE has been utilized in titrating energy delivery.^16–18^ Because of its immediate imaging acquisition, ICE has proven invaluable in managing complications such as hemopericadium and in avoiding the onset of atrioesophagheal fistula or coronary artery injury.^19^ The role of ICE has increased in the mapping and ablation of PM VT. Initial reports by Yamada et al. suggest that PM VT may be challenging to ablate given the complex anatomical substrate involved.^20^ Seiler et al. further described the potential role of ICE in identifying and recognizing the PM arrhythmogenic focus.^21^ The PMs themselves are also part of a complex architecture with the chordae, and a prior report of ablation at the tip of the left ventricular PM guided by ICE has been described.^22^ Given the complex nature of PM anatomy, ICE has proven to be invaluable in the real-time assessment of catheter contact and stability. Patients with suspected PM VT, especially with prior failed ablations, would likely benefit from ICE. Aside from assessing anatomy and catheter contact, ICE can provide real-time feedback regarding ablation effect. As shown in our case, changing from NS to HNS led to a marked increase in the echogenicity of the area of interest, which can provide an instant qualitative assessment of lesion effect.

Our case also highlighted the role of respiratory manipulation in order to promote improved catheter stability/contact. Used occasionally during left atrial appendage closure and percutaneous epicardial access, apnea can reduce another level of cardiac motion created by diaphragmatic movement during the respiratory cycle. Clues to catheter displacement due to respirations include local electrogram changes or cyclical changes in catheter force that revert back to “baseline” with each breath. Given the complex nature of the PMs, the role of induced apnea in these cases warrants further study. Aside from complete apnea, high-frequency jet ventilation (HFJV) is a method of ventilation that can reduce respiratory movement to near-static conditions. HFJV is aimed at providing effective gas exchange with minimal tidal volumes and, consequently, minimal cardiac and chest wall movement. This enhances catheter stability and precision during minimal invasive procedures and is useful in ventricular and atrial tissue ablation.^23,24^ A recently published article showed a significantly improved success rate of ablations for patients undergoing atrial fibrillation ablation with HFJV (31% versus 50%; p = 0.012). Further, investigators reported a significant improvement was achieved in the maintenance of a level of contact force of more than 5 g and a higher mean force variability index was realized with HFJV in comparison with standard ventilation (p ≤ 0.001), facilitating improved lesion delivery and ablation-line integrity.^25^ Prior to this study, Kumar et al. demonstrated the benefit of catheter stability afforded by regulating ventilation and reported significant improvement in the average force–time integral during apnea as compared with standard ventilation.^26^ While these studies focused on atrial ablations, the conceptual experience is potentially applicable to ventricular ablations as well, although we are unaware of any trials using the principle of HFJV during VT ablation published at this time.

Other adjunctive measures that were not employed but were described include bipolar RF ablation, ethanol injection, bipolar needle injection, an open surgical approach, and even radiation therapy. Given the complex anatomy of the PM/chordae apparatus, we felt that obtaining appropriate catheter positioning for bipolar RF was challenging. Further, the intramural PM focus precluded ethanol injection into a specific coronary venous or arterial branch and may also lead to an excessive ablation effect, leading to PM dysfunction. Bipolar needle injection and mapping, which would allow for intramural activation mapping and perhaps a more targeted ablation, have been described. This may warrant, however, multiple injections within the PM complex. Further, the intramural focus lowered the utility of an open surgical approach, while radiation therapy may lead to collateral damage capable of lasting for decades in this young patient.

Cryoablation might also have been considered given the complexity of the PM architecture and need for stability. Prior reports have suggested its role in PM VT ablation and it could have been considered in this situation.^27^ Given the concern regarding the need for increased lesion depth, however, this was not performed before considering HNS. Finally, percutaneous epicardial mapping was not performed. This was primarily due to the completion of prior epicardial mapping that did not reveal significantly early electrograms and the performance of unsuccessful empiric ablation in this region. It is possible, however, that repeat epicardial high-density mapping with multipolar catheters may have led to the elucidation of a position closer to the true site of origin.

## Conclusion

We describe a unique case of a young patient who presented with a nearly incessant PM VT despite prior (failed) ablations that warranted multiple adjunctive techniques using the high-impedance irrigation properties of HNS in combination with the catheter stability insurance provided by direct ICE imaging and respiratory control. In this case, there were no complications and the patient has remained free from PVCs for more than six months. Further research is needed to understand the optimal situations necessitating the use of these techniques during ablation for ventricular arrhythmias.
